# An inventory of biodiversity data sources for conservation monitoring

**DOI:** 10.1371/journal.pone.0242923

**Published:** 2020-12-02

**Authors:** P. J. Stephenson, Carrie Stengel

**Affiliations:** 1 IUCN SSC Species Monitoring Specialist Group, c/o Laboratory for Conservation Biology, Department of Ecology & Evolution, University of Lausanne, Lausanne, Vaud, Switzerland; 2 Global Wildlife Conservation, Austin, Texas, United States of America; Swansea University, UNITED KINGDOM

## Abstract

Many conservation managers, policy makers, businesses and local communities cannot access the biodiversity data they need for informed decision-making on natural resource management. A handful of databases are used to monitor indicators against global biodiversity goals but there is no openly available consolidated list of global data sets to help managers, especially those in high-biodiversity countries. We therefore conducted an inventory of global databases of potential use in monitoring biodiversity states, pressures and conservation responses at multiple levels. We uncovered 145 global data sources, as well as a selection of global data reports, links to which we will make available on an open-access website. We describe trends in data availability and actions needed to improve data sharing. If the conservation and science community made a greater effort to publicise data sources, and make the data openly and freely available for the people who most need it, we might be able to mainstream biodiversity data into decision-making and help stop biodiversity loss.

## Introduction

Biodiversity continues to decline [1, 2]. There is growing demand for more evidence-based conservation, with data informing decisions and evaluating performance [3, 4]. This will become even more important as the world commits to post-2020 biodiversity targets [5]. In addition to governments and civil society organisations, businesses striving for sustainability also struggle to identify suitable indicators and data sets [6]. Effective, evidence-based biodiversity conservation and natural resource management requires data on the state of species and habitats, the threats and pressures they face, and the policies and actions implemented to address them. However, although monitoring is standard best practice in project management, often it is not conducted thoroughly [7]. As a result, biodiversity data are scattered, fragmented, a challenge to assemble, and rarely available to decision makers [8–10].

Blockages to biodiversity monitoring include lack of access to existing data sets [9], exacerbated by many organisations acting independently to develop their own databases and data platforms [11]. In high-biodiversity countries, such as in tropical Africa, limited capacity and expertise for data sharing and use are often compounded by more limited resources to pay for raw images and data processing, as well as limited internet capacity [9, 12, 13]. Many of the assessments of African biodiversity data have been led and conducted by scientists who are predominantly based outside the region [14, 15], reflecting more systemic issues with capacity for research and monitoring [16–18]. It is therefore perhaps unsurprising that all of the global data sets, and most of the scientists with access and capacity to analyse them, are housed in Europe and North America.

Governments, NGOs and international organisations monitor delivery of global conservation goals, such as the Aichi Targets and the environment-focused Sustainable Development Goals, by using a number of key global databases, such as the IUCN Red List of Threatened Species [19], Protected Planet [20] and the Living Planet Index [21]. The data in these databases can be used to track biodiversity at local, national and global scales [4, 22, 23]. But these databases focus on the state of biodiversity, mostly vertebrates, and on one conservation response (protected areas). What other data are available that could be used by conservation agencies (e.g. government departments, NGOs) and other natural resource managers (e.g. businesses, local communities) at national or international level to enhance biodiversity monitoring?

Only limited efforts have been made to date to identify and share available biodiversity data sources, and these have focused on a single theme, such as threats [24], or one region, such as Europe [11], and they did not target data specifically of use for monitoring. We therefore undertook an inventory of existing global data sets, databases and data platforms, as well as reports that synthesise such data, in order to determine data sources that could help conservationists and natural resource managers monitor biodiversity, especially the state of species and habitats, the threats and pressures they face, and conservation responses. This represents the first attempt to summarise data sources specifically relevant to biodiversity monitoring.

## Materials and methods

Data sources and reports were identified through a web search and literature review using a combination of key terms (e.g. biodiversity, conservation, data, data set, database) and terms linked to key threats and actions (e.g. bycatch, deforestation, offtake, pollution). A snowballing technique was used to source other literature from that uncovered. In addition, websites of key organisations working on biodiversity data were explored (e.g. those of the Biodiversity Indicators Partnership, GEOBON, UN agencies such as FAO and UN Environment, and UNEP-WCMC). Similarly, the latest versions of key biodiversity reports were mined for references to specific data sets, especially the Global Biodiversity Outlook, Global Environment Outlook, and assessments of the Intergovernmental Platform on Biodiversity and Ecosystem Services (IPBES).

While an effort was made at the start of the assessment to differentiate between data platforms (websites and tools used as portals to access multiple data sets) and other data sources, this proved infeasible, largely because almost all organisations compiling data sources at the global level use data acquired from others. Therefore, throughout the inventory, the term data source is used to cover data sets, databases and data platforms, whether they are primary or secondary sources.

Data sources were included in the inventory if they met the following criteria: potential relevance for monitoring biodiversity state, pressures and responses at the global level by key stakeholders (governments, international organizations, civil society and NGOs, conservation agencies and businesses striving for sustainability); at least some time-series data or plans to collect time-series data; up to date (with data added in at least the last 5years or so). Regional and other sub-global level data sources were excluded. The most useful data sources would have large volumes of widespread data that were scalable, of high quality and regularly updated, but it was not always easy to judge how these criteria were met from the information available. It was also not always easy to assess how accessible data were and if they were free-of-charge. Indeed, even if data are freely accessible they are not always easy to use [24]. Therefore, we note that some data sources in the inventory may still prove challenging for monitoring purposes when investigated in more detail.

Multiple, linked data sets managed by a single agency and consolidated in a single database were considered as one source. For example, the FAO Fisheries and Aquaculture database has numerous data sets (e.g. Global Production, Global Capture Production, Global Tuna Catches by Stock, Global Aquaculture Production, Atlas of Tuna and Billfish Catches, Global Number of Fishers, Consumption of Fish and Fishery Products, etc.) but was counted as one fisheries data source. Similarly, habitat and land cover data were not disaggregated by remote sensing data set but, instead, we identified data sources that consolidated from different sets. For example, forest cover data sources are numerous (e.g. Global Forest Change, Global Forest Watch) but all in turn source their data from a variety of remote sensing programmes, such as ESA’s GlobCover and NASA’s Landsat. The intent was to identify wherever possible pre-prepared data of use to managers, not raw data files for GIS analysis.

Data sources were clustered based on their uses for monitoring biodiversity state, biodiversity threats and pressures, and conservation responses, as per the Pressure-State-Response indicator model widely used in biodiversity conservation monitoring [4, 7, 25]. The data sources that could potentially be used to monitor pressures and responses were clustered under the threats and conservation actions categories developed by IUCN and the Conservation Measures Partnership, CMP [26]. For this exercise, the newer, revised versions of both classifications were used: the CMP Direct Threats Classification (v 2.0 of 2016) and the CMP Conservation Actions Classification (v 2.0 of 2016) [27]. In each case, we developed examples of related indicators and indicator categories associated with each action and each threat and then listed examples of global databases and platforms of potential use in monitoring them. Note that, in some cases, a database could be used for more than one indicator. For example, forest cover data can show the state of the habitat, but a trend in forest cover can also monitor a trend in forest loss, which is a threat. Note too that state metrics–such as species populations trends and habitat cover–can also be used to monitor the impact of various threats (e.g. deforestation rates in relation to infrastructure development or mining).

## Results, conclusions and discussion

**Fig 1:**
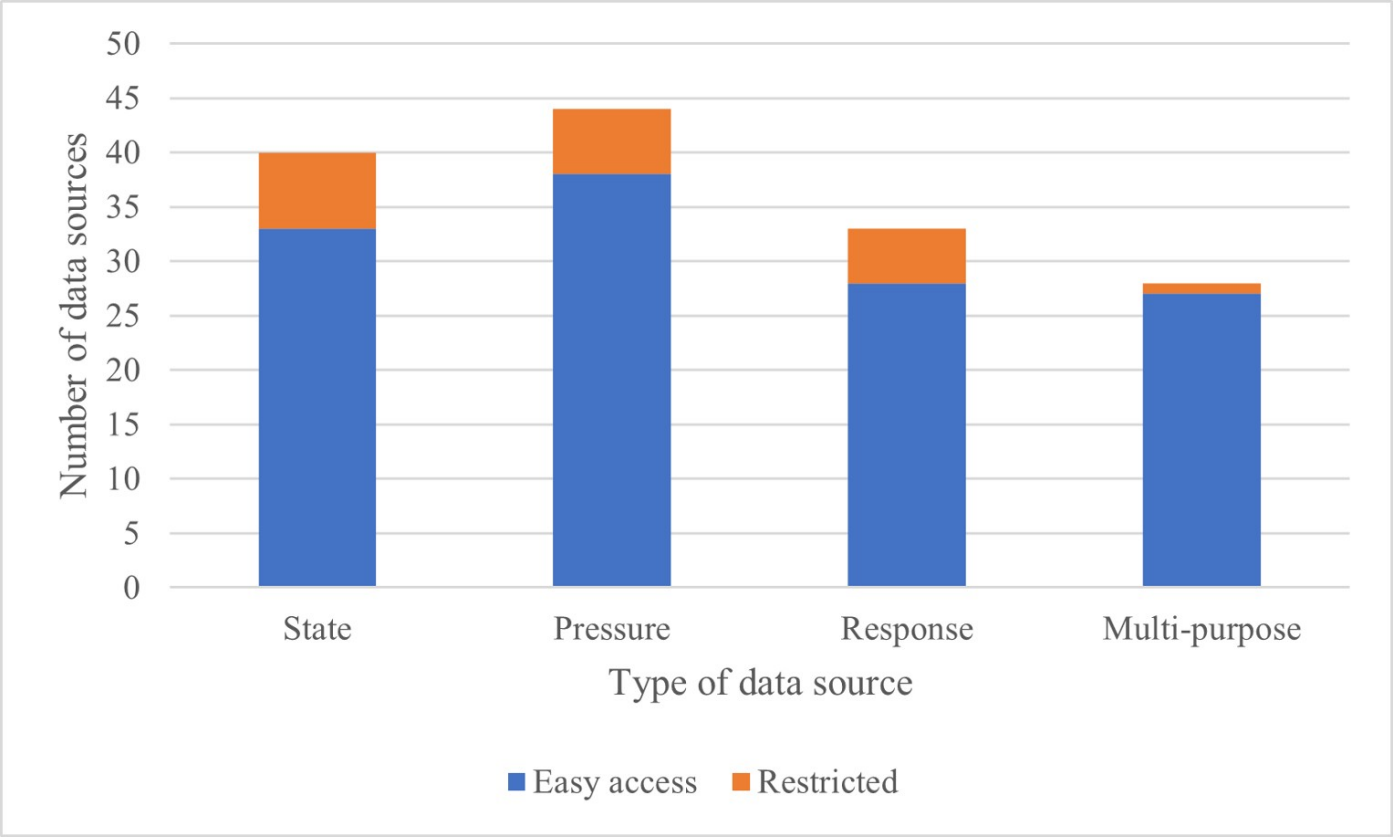
Data sources identified. A summary of the 145 data sources identified by data type, and the proportion known to have at least some data easily (i.e. instantly, openly and freely) accessible.

A total of 145 global data sources were identified of potential value in conservation monitoring (Fig 1): 40 data sources for biodiversity state, with at least 33 open access (S1 Table); 44 data sources for biodiversity pressures and threats, with at least 38 open access (S2 Table); 33 data sources for conservation responses, with at least 28 open access (S3 Table); and 28 data sources that cut across multiple indicator types, with at least 27 open access (S4 Table). Twenty-six types of report were identified (S5 Table) that regularly synthesise biodiversity data from diverse sources. These helped identify some of the data sources. The shortlist of data sources that, from this preliminary analysis, appear to be the most useful in accessing data for monitoring biodiversity were compiled (Table 1).

**Table 1:** Summary of key data sources for monitoring biodiversity and their potential uses for conservation agencies.

Lead agency	Data source	Useful data available	Potential uses for conservation agencies
BirdLife International	World Database on Key Biodiversity Areas (KBAs)	Coverage of KBAs and key species within them	Map KBAs in the agency’s priority countries/ecoregions and overlay with protected areas and threatened species distributions to identify priority sites; Monitor proportion of KBAs protected over time.
FAO–UN Food and Agriculture Organisation	Fisheries and Aquaculture database	Multiple fisheries datasets. Includes volume of fish catches landed by country or territory, by species or a higher taxonomic level, by FAO major fishing areas, and year for all commercial, industrial, recreational and subsistence purposes.	Monitor fisheries offtake in any marine sites of interest to the agency or for any target fish species.
IUCN–International Union for Conservation of Nature	IUCN Red List of Threatened Species	Geographical range, estimated population trends (or relative abundance), habitat use, life history traits, use and trade, threats, conservation actions in place and conservation actions needed	Create a Red List Index for the agency’s priority countries or track the status of priority species.
NOAA—US National Oceanic and Atmospheric Administration	Coral Reef Watch	Daily global 5km satellite Coral Bleaching Heat Stress Alert Area	Identify (daily) marine sites at highest risk of coral bleaching.
UNEP-WCMC (UN Environment World Conservation Monitoring Centre)	CITES Trade Database	Number of individual plants and animals traded, exporting and importing countries	Track legal or illegal trade in species relevant to the agency, (perhaps displayed like the UNEP-WCMC Big Cat Trade Dashboard).
	Global Database on Protected Area Management Effectiveness	Protected area management effectiveness	Monitor the effectiveness of protected areas, showing improvements over time as a result of the agency’s support; Compare management effectiveness across different categories of PA to highlight capacity gaps.
	IBAT—Integrated Biodiversity Assessment Tool	Threatened species distribution and status in relation to KBAs and protected areas; proximity of business activities to threatened species, KBAs and PAs.	Map KBAs in the agency’s priority countries/ecoregions and overlay with protected areas and threatened species distributions to identify priority sites; Monitor proportion of KBAs or key threatened species range protected over time.
	Protected Planet–World Database on Protected Areas	Protected area status and coverage	Map protected areas of interest and overlay species data.
	Wetlands Extent Trends	Trends in wetland area over time	Show trends in wetland area over time in priority agency sites/countries/ecoregions.
University of British Columbia	Marine Trophic Index	Fishing levels in economic zones by country and by fish taxa; time-series data on MTI for large marine ecosystems	Monitor the Marine Trophic Index of the agency’s priority seascapes to assess trends in the impacts of fishing (down the food web).
Wetlands International	International Waterbird Census Database	Waterbird populations (distribution and abundance)	Show trends in waterbird populations in the agency’s priority sites/countries/ecoregions.
World Resources Institute	Climate Watch	Greenhouse gas emissions; climate risk, vulnerability and readiness scores	Show trends in national, regional or global emissions and climate risks.
	Global Forest Watch	Forest cover changes, carbon stored and emitted from forests, active fires and (in develop-ment) commodity supply chain impacts on forests.	Monitor forest cover changes in the agency’s priority sites/countries/ecoregions; the map function could show deforestation frontiers.
			Active fires could also be mapped for the agency’s areas of interest.
	Resource Watch	Access to datasets relating to Cities, Climate, Energy, Food, Forests, Ocean, Society, Water	The agency could become a WRI partner to help develop and use the platform, enhancing the biodiversity elements and their value to conservationists
Zoological Society of London	Living Planet Index	Time-series data for populations of mammal, bird, fish, reptile and amphibian species	Create an index of trends in vertebrate populations in the agency’s priority sites/countries/ecoregions or track the population levels of priority species.

Conservation agencies are government, community and NGO entities or projects, especially those working in high-biodiversity countries. Data sources would also be relevant for some businesses striving for sustainability. All data sources listed are openly accessible or available on request (although IBAT requires a paid subscription for business users).

While 86.9 per cent of the data sources allowed instant, open and free access to at least some of their data, the usefulness of that data and the relevance of the formats was not ascertained. The degree to which it was possible to assess what data are available and accessible, the origins of the data, the precise indicators the data measure, and the length of any time series available was very variable and the information was often complicated to find. In some cases, even the organisations managing the data or the websites where data could be located were difficult to identify easily.

Categorising (or clustering) data sources was challenging. Clumping data under broad terms such as “state” was too general, and further subdivisions might be useful (perhaps based on taxa or biomes or geography). On the other hand, the CMP classifications for threats and actions were too detailed to make clustering under each individual action or threat a meaningful (or sometimes feasible) exercise. Once the new post-2020 biodiversity targets have been finalized to replace the Aichi Targets, we will try to cluster some of the data sources under each new target and its associated indicators (sensu [28]) Categorising sources was further complicated by the fact several databases contain multiple data sets (the FAO Fisheries and Aquaculture database being a good example) so can also be listed under several data categories (state, pressures/threats or responses/actions).

Key actors involved in managing data sources of most use to biodiversity monitoring include BirdLife International, the Secretariat of the Convention on International Trade in Endangered Species of Wild Fauna and Flora (CITES), the UN Food and Agriculture Organisation (FAO), the International Union for Conservation of Nature (IUCN), the US National Oceanic and Atmospheric Administration (NOAA), UN Environment World Conservation Monitoring Centre (UNEP-WCMC), University of British Colombia (the Sea Around Us), Wetlands International, the World Resources Institute (WRI), and the Zoological Society of London (ZSL). This reflects earlier analyses of data sets which noted that a small number of agencies curate most of the data. For example, Joppa et al [24] found six data providers account for more than a fifth of biodiversity threat data sets.

Global data sources exist that are useful in providing time-series data of biodiversity measures such as vertebrate population trends, habitat cover trends, species extinction risk, protected area coverage and management effectiveness, fisheries and climate, though the frequency the data sets are updated varies and there are gaps in taxonomic and geographic coverage. Overall, across data sources, there was better coverage of some biomes, taxa, threats and responses than others. For example, vertebrates (especially large mammals and birds), forests, protected areas, fisheries and certain aspects of climate change (greenhouse gas emissions, sea temperature) are better represented in the data sources than other forms of biodiversity information. There are more data for terrestrial biomes than for marine or freshwater. Even for well-studied taxa like birds, time-series data are mostly from Europe and North America. These findings reflect recognised global trends, with major gaps in biodiversity data in high-biodiversity tropical countries [13, 29, 30]. There are generally more data for Europe and North America than other regions, and more for terrestrial birds, mammals and trees than for other taxa, but there is a lot of local variation [4, 29, 31–34].

There was an uneven spread of databases across CMP pressure categories, with a disproportionately large number for climate change and biological resource use and none that were relevant or easily accessible for energy production & mining and human intrusions & disturbance. For responses, there were numerous data sources relating to livelihood, economic & moral incentives and conservation designation & planning, but none for awareness raising, law enforcement & prosecution, and education & training. There are limited data at global level that allow direct measurement of the benefits and livelihood gains accrued by people due to conservation. This may partly be an issue of lack of data collection [35], but may also reflect the fact local data from site-level projects are not easy to aggregate to regional or global scales.

Only data from satellite-based remote sensing is updated on a daily basis (and therefore provides almost real-time monitoring). WRI’s Global Forest Watch updates information every day on forest loss and active fires. NOAA’s Coral Reef Watch updates data daily on sea temperature, flagging coral reefs where bleaching is most likely.

Some data sources provide access to existing maps (e.g. SEDAC). However, there appears to be a growing trend for organisations to produce portals where people can click on different data sets to make them appear as overlays on a world map (examples include Global Forest Watch, Map X, OBIS-SEAMAP). In most instances, however, mapping has limited use for monitoring, primarily due to the lack of scope to track trends over time in quantifiable ways. It was also often difficult to ascertain how easy it is to access the source data behind the maps.

There are about 15 data sources, with regularly updated time-series data, that were considered to have the most potential for helping conservation agencies with biodiversity monitoring (Table 1). These data sources are easily (if not always instantly or freely) accessible and provide data on key issues including coral bleaching, fisheries, forest cover, greenhouse gas emissions, Key Biodiversity Areas, protected areas, species distribution and abundance, wetlands extent and wildlife trade. However, for the full data landscape to become more manageable and accessible to people who need it most, and to provide all the types of information required, several actions need to be taken by conservationists, scientists and other end users.

### What needs to happen next

#### Define terms and relationships

An ontology of databases, data sets, data platforms, data portals and data sources relating to biodiversity would help clarify types of data available and their interlinkages. The system should clarify which elements of biodiversity monitoring (state, pressure, response) the data are, can or could be used for. The CMP/IUCN threat and response categories can help to some extent, although they may need adapting for the monitoring context by selecting those most relevant to different scales (e.g. highlighting that data on direct benefits to people may be best collected at local levels rather than global levels).

Existing thinking around data classifications should be built on. For example, Bingham et al. [11] identified three types of elements in the bioinformatics landscape: elements with a single specific focus (such as taxonomic backbones); higher-level elements that rely on one or more other elements (e.g. AquaMaps, which harvests species occurrence data from the Global Biodiversity Information Facility, GBIF, and the Ocean Biogeographic Information System, OBIS, and life-history parameters from FishBase); and complex elements that rely on several other elements (e.g. the EMODnet Portal, which can combine multiple datasets in interactive maps, drawing on data sets from, for example, the European node of OBIS, the CITES Trade database, and the IUCN Red List of Threatened Species). This framework could be integrated with the approach taken by Konig et al. [36] who conceptualised widely-used biodiversity data types according to their domain (the aspect of biodiversity that is described) and informational resolution (how specific the description is). Such an ontology would help in refining the inventory of data sources and help clarify the numbers and types of sources available. For example, an assessment of data sets looking at threats ([24]; S1 Table) considered multiple data sets looking at the same issue (e.g. forest cover) or multiple versions of the same data set (e.g. GlobCover 2.2 and GlobCover 2.3) or disaggregated data from one agency (e.g. FAO data on different types of livestock–buffalo, cattle, goats, sheep, pigs, poultry, etc.). The current study lumped data sources for efficiency of use and analysis. An ontology would therefore help in comparing different data source inventories.

As well as developing an ontology of data source types, existing data sources should strive to make it clearer (preferably on the homepage of their websites) which data they are sourcing and from where, and if it is instantly, openly and freely available.

#### Address data gaps and biases

Conservation practitioners and scientists, as well as other biodiversity data users in governments., NGOs and businesses, need to identify and systematically fill data gaps. All stakeholders should, at the very least, be collecting primary data to monitor their biodiversity priorities and projects. In addition, efforts need to be made to focus on data collection in the tropical regions housing most of the species and on those taxa that are most under-represented in data sets, such as marine and aquatic species, small mammals, invertebrates, fungi, plants and wide-ranging species [4, 13, 29, 32, 34, 37, 38]. More data from tropical countries are also needed if we are to be able to predict the impacts of climate change on tropical species [39] and assess projected range to set conservation priorities [40]. Even the larger and more widely known and used global data sets, such as GBIF, the Living Planet Index and the IUCN Red List of Threatened Species, tend to have data skewed taxonomically and geographically [29, 32, 37], and more detailed breakdowns of gaps would help focus data collection efforts [41]. As well as data on the state of biodiversity and conservation responses, threat monitoring needs to become more mainstreamed into conservation projects [42].

Data need to be collected regularly and efforts made (at least in some cases) to come as close to possible to real-time monitoring. Satellite-based remote sensing data is becoming cheaper and more easily available [43], suggesting real-time monitoring of biodiversity from space (especially the state of, and loss of, key habitats) will continue to expand. Technological advances have ensured that satellite-based remote sensing is increasingly complemented by the newest generation of Earth-based sensors [13, 44], including camera traps [45], acoustic recording devices [46] and unmanned aerial vehicles or drones [47], as well as by environmental DNA monitoring [48]. Some projects are also underway to provide more integrated monitoring systems. For example, the conservation agency Resolve, with its NGO partners like the National Geographic Society and the Zoological Society of London, and its technical partners like Google and Microsoft, are working to try to establish a network of space-based and Earth-based sensors in key conservation areas that will lead to real-time monitoring of species, habitats and illegal activities [49]. Therefore, we may not be far away from having live-streamed updates from key conservation areas.

Although remote sensing offers many opportunities for enhancing biodiversity monitoring [44], current technology tends to bias data collection towards trees, large mammals and birds. While, in many instances these may be good indicators of broader ecosystem health, a more complete understanding of biodiversity trends is likely to come only from having human observers on the ground in key sites [30, 44, 50]. Recently developed monitoring protocols that include oft-neglected taxa like plants and invertebrates (e.g. [51, 52]), for example, tend to rely on methods that require a human presence in the field.

Data that are collected need to be shared as widely as possible by uploading them into national, regional and global databases [50]. Databases that are not updated regularly with new data will soon become less useful [53]. Data quality and reliability is also key [54]. To be useful for monitoring, data need to be freely available, of a suitable spatial resolution, up to date, repeated and assessed for accuracy, yet only 5% of data sets on biodiversity threats meet these criteria [24] and other data sources are not likely to be better.

#### Share data and build capacity where it is needed most

There have been numerous calls for greater efforts to build capacity for data collection and use where it is most needed in high-biodiversity countries [9, 30, 55, 56]. Capacity for data use will be much enhanced by making existing data available to conservationists, such as protected areas managers, and other end-users such as businesses. This will entail letting them know what is available, where to find it and how to access it. To lead the way we will share our full database of data sources—a combination of the tables presented in this paper along with S1–S5 Tables. From December 2020, the database will be hosted by the IUCN SSC Species Monitoring Specialist Group on its data and databases webpage (https://www.speciesmonitoring.org/data_ sources.html), with a link to the page from the Global Wildlife Conservation website. Over time, details of each data source will be elaborated and its uses explored and explained in more detail. The database will be updated regularly and made freely available as a resource for conservationists, scientists and other data users and decision makers. We will also use our networks to disseminate the database widely in high-biodiversity countries. Database managers need to help too. A recent review of European data sets found only around a third of data-providers provides unrestricted data access [57]. Even if data are technically free to download, there are often various hoops to jump through (e.g. individuals or committees that need to approve access) or the data are not in a format known or usable to the recipient. Some data sources charge for certain uses, such as corporate access. A study to determine which funding models work best for maintaining databases and making them as openly accessible as possible would be apposite.

Impediments to biodiversity data sharing include lack of professional recognition of scientific data publishing efforts [58], compounded by a lack of infrastructure for easy data sharing [59]. Therefore, it is necessary “to motivate and reward the contribution of data to international integrated databases by bringing such data into the mainstream of respected scientific publication” [60]. This will involve the development of “mechanisms for data citation and indices of data access comparable to those for citation systems in print journals” [61]. The IUCN Red List of Threatened Species [19] sets a good example, where each assessment is saved and allocated a DOI (digital objective identifier) to make the data for a given species a citable publication. Several other databases, such as the Living Planet Index, also encourage data contributions through their website. Other options include working towards the mainstreaming of data papers by making the publishing of data mandatory in research project proposals and performance assessments [62], and adopting standards related to data citation, accessibility, metadata, and quality control in order to facilitate integration of data across data sets [60, 63, 64].

Data will also be made more accessible if data sets are fused or integrated (see, e.g., [65]) and more effort is made to integrate non-Western data sources into biodiversity databases [66]. We conclude by repeating the call of Stephenson et al. [30] for all actors “to collaborate in harmonising databases and platforms and in enhancing interoperability and version control between them”. This will involve using Application Programming Interfaces (APIs) to enhance connections and interoperability between databases.

#### Conservation agencies need to use data

Conservation agencies, as well as other natural resource managers such as governments, businesses and NGOs, can integrate global data sets into their own monitoring systems to complement site-level, in situ data collection [4], to measure metrics of institutional impact [7, 67] and to enhance impact evaluation [68]. More agencies need to use such data, complementing it with their own locally-collected data from projects to monitor progress. This can then replicate at the programme or institution level the same trends in linked indicator sets that are analysed at the global level (e.g. [31, 69]), providing a conservation narrative around increases in pressures and declines in biodiversity state (and often also a decline in ecosystem services and other benefits for people). Further examples of how data can be presented and visualised around a narrative can be found in many of the global status reports (see S5 Table), especially Global Biodiversity Outlooks, Global Environment Outlooks and Living Planet Reports. Stories can be told on, for example: the impacts of commodities on forests (by showing data on commodity production alongside forest cover change for a site, landscape, country or region); the impacts of wildlife trade on species populations (by showing data on species population trends, alongside data on illegal killing and illegal trade–especially in species such as elephants, rhinos and tigers at national or global scales); the impacts of climate change on reefs (by showing data on greenhouse gas emissions alongside average sea temperatures alongside coral cover); and the impact of overfishing on ocean health (by showing catch data alongside the Marine Trophic Index or Ocean Health Index).

Ultimately data need to be used in decision-making, and this can be enhanced by presenting data in formats that are easy to interpret, such as graphs, maps and dashboards [4, 7, 70].

#### Work together

Conservation agencies, data providers and database managers need to break out of institutional silos and move away from a focus on agency-specific databases and platforms to collaborate more on coproducing and sharing data. Our inventory shows a huge level of duplication of effort, with different organisations often developing similar data sources or data mapping platforms to each other. This must result in widescale cost inefficiencies and, in effect, the wasting of valuable conservation funding. Concepts such as k*nowledge coproduction* (the collaborative process of bringing diverse knowledge sources and types together to address a defined problem; [71]) and co-construction between knowledge and action [72] should be applied more widely to biodiversity conservation. Identified gaps between different data managers and users will only be bridged through improved co-ordination and collaboration [9, 72]. Local and international NGOs, as well as academia and businesses, have a significant role to play in supporting government agencies and it is encouraging to see that several global efforts to improve biodiversity monitoring explicitly target high-biodiversity countries [30, 41]. Some of the large biodiversity databases could be useful tools for business throughout project planning and implementation [6, 73], so businesses could in turn share data of use to resource-strapped governments and contribute more to the costs of data source maintenance.

## Conclusion

The lack of access to biodiversity data is a major impediment to adaptive management in conservation and natural resource management. Managers need to continue to collect primary data for their projects and programmes to monitor progress but, in many cases, their efforts will be enhanced if they can access and use data on biodiversity pressures, states and responses from global sources. Numerous data sources exist and we have identified a preliminary list of some of the potentially most useful, but many are difficult to find, access or use easily. However, if the conservation, science and business communities could make a greater effort to share and publicise data sources and make existing tools and data freely available for the managers who most need them, we might be able to mainstream biodiversity data into decision-making and ultimately stop biodiversity loss.

## Supporting information

S1 Table Global biodiversity data sources of potential value in monitoring biodiversity state. Those data sources where at least some data (or mapping of data) appear to be instantly, freely and openly available are flagged with a star (*). Those data source on priority sites (AZE sites, G200 ecoregions, KBAs, etc) will usually need to be used in tandem with other data sources to be relevant to monitoring certain indicators (e.g. number/abundance of threatened species in priority areas). Note that some data sources would need updating before they could be of use. An updated list, with additional information, will be posted on https://www.speciesmonitoring.org/data-sources.html. Data source managers are encouraged to send any additional information or updates to SpeciesMonitoringSG@gmail.com.
(DOCX)

S2 Table Global data sources of potential value in monitoring pressures and threats to biodiversity. Those data sources where at least some data (or mapping of data) appear to be instantly, freely and openly available are flagged with a star (*). Note that some data sources would need updating before they could be of use. An updated list, with additional information, will be posted on https://www.speciesmonitoring.org/data-sources.html. Data source managers are encouraged to send any additional information or updates to SpeciesMonitoringSG@gmail.com.
(DOCX)

S3 Table Global data sources of potential value in monitoring conservation responses to biodiversity loss. Those data sources where at least some data appear to be freely and openly available are flagged with a star (*). Note that some data sources would need updating before they could be of use. An updated list, with additional information, will be posted on https://www.speciesmonitoring.org/data-sources.html. Data source managers are encouraged to send any additional information or updates to SpeciesMonitoringSG@gmail.com.
(DOCX)

S4 Table Global data sources with multiple uses for biodiversity monitoring. Those data sources where at least some data seem to be freely and openly available are flagged with a star (*). Note that some data sources would need updating before they could be of use. An updated list, with additional information, will be posted on https://www.speciesmonitoring.org/data-sources.html. Data source managers are encouraged to send any additional information or updates to SpeciesMonitoringSG@gmail.com.
(DOCX)

S5 Table Reports synthesising large biodiversity data sets over time. (DOCX)
