# Supplementary material for: An inventory of biodiversity data sources for conservation monitoring
Source: PLoS One. 2020 Dec 2;15(12):e0242923. doi: 10.1371/journal.pone.0242923 (PMC7710106; doi:10.1371/journal.pone.0242923)
Supplement: S1 Table — Those data sources where at least some data (or mapping of data) appear to be instantly, freely and openly available are flagged with a star (*). Those data source on priority sites (AZE sites, G200 ecoregions, KBAs, etc) will usually need to be used in tandem with other data sources to be relevant to monitoring certain indicators (e.g. number/abundance of threatened species in priority areas). Note that some data sources would need updating before they could be of use. An updated list, with additional information, will be posted on https://www.speciesmonitoring.org/data-sources.html. Data source managers are encouraged to send any additional information or updates to SpeciesMonitoringSG@gmail.com. (DOCX) [file pone.0242923.s001.docx]

**S1 Table. Global biodiversity data sources of potential value in monitoring biodiversity state.** Those data sources where at least some data (or mapping of data) appear to be instantly, freely and openly available are flagged with a star (*). Those data source on priority sites (AZE sites, G200 ecoregions, KBAs, etc) will usually need to be used in tandem with other data sources to be relevant to monitoring certain indicators (e.g. number/abundance of threatened species in priority areas). Note that some data sources would need updating before they could be of use. An updated list, with additional information, will be posted on https://www.speciesmonitoring.org/data-sources.html. Data source managers are encouraged to send any additional information or updates to SpeciesMonitoringSG@gmail.com.

| **Data source** | **Lead agency** | **URL** | **Description** |
| --- | --- | --- | --- |
| Alliance for Zero Extinction Sites | Alliance for Zero Extinction | <https://zeroextinction.org/> | Information on AZE sites and the species they contain, each of which is the last remaining refuge of one or more Endangered or Critically Endangered species as defined in the IUCN Red List of Threatened Species. Summary data in maps; full data available on request. |
| AmphibiaWeb* | University of California, Berkeley | <https://amphibiaweb.org/index.html> | Information on amphibian declines, natural history, conservation, and taxonomy. |
| Aqua Maps* | FishBase and SeaLifeBase | <https://www.aquamaps.org/search.php> | Generates model-based, large-scale predictions of natural occurrences of marine and aquatic species. Derived from GBIF, OBIS, FishBase, SeaLifeBase & AlgaeBase. |
| BioCASE (Biological Collection Access Service)* | Botanic Garden and Botanical Museum, Berlin | <http://www.biocase.org/index.shtml> | Links together specimen data from natural history collections, botanical/zoological gardens and research institutions worldwide to identify historical sites of species occurrence. |
| Biodiversity Habitat Index | CSIRO | <https://www.bipindicators.net/indicators/biodiversity-habitat-index> | Translates the observed spatial distribution of habitat loss and degradation into expected impacts on retention of terrestrial biodiversity. Data available on request. |
| Birdlife Datazone* | BirdLife International | <http://www.birdlife.org/datazone/home> | Distribution and abundance of bird species worldwide, mostly presented as content of IUCN Red List. Population data only show general trend (as per Red List). Distribution maps need to be requested. |
| CGMFC-21: Continuous Global Mangrove Forest Cover for the 21st Century* | Salisbury University | <http://faculty.salisbury.edu/~sehamilton/mangroves/> | Mangrove cover annually; needs updating. |
| DEIMS-SDR (Dynamic Ecological Information Management System - Site and dataset registry)* | Centre for Ecology and Hydrology, Natural Environment Research Council, UK | <https://deims.org/> | Data from long-term ecosystem research sites. |
| Domestic Animal Diversity Information System (DAD-IS)* | FAO | <http://www.fao.org/dad-is/dataexport/en/> | Data on domestic strains of animal per country for monitoring Aichi Target 13. |
| eBird* | Cornell Lab of Ornithology | <http://ebird.org/> | Citizen science bird observations. |
| eMammal* | Smithsonian | <https://emammal.si.edu/> | Data management system and archive for camera trap research projects. |
| Ecologically or Biologically Significant Marine Areas* | Secretariat of the Convention on Biological Diversity | <https://www.cbd.int/ebsa/> | As with KBAs and protected areas, the use of these area in monitoring is to identify sites of importance for conservation. |
| Ecosystem (or Ecoregion) Intactness Index* | University of Queensland | <https://espace.library.uq.edu.au/view/UQ:f51cace> | Intactness scores for the world's terrestrial ecoregions; needs updating. |
| EDGE Database | Zoological Society of London | <https://www.edgeofexistence.org/search/> | Data available on request on species considered Evolutionarily Distinct and Globally Endangered (EDGE), such as population numbers and trends, range, threats, etc. Currently focused on vertebrates and corals. |
| FishBase* | FishBase consortium | <http://www.fishbase.org/> | A global biodiversity information system on finfishes: taxonomy, biology, trophic ecology, life history, and uses, as well as historical data reaching back to 250 years. |
| Global 200 Ecoregions* | WWF-US | <https://www.worldwildlife.org/publications/global-200> | Data on 238 terrestrial, freshwater, and marine ecoregions that harbour exceptional biodiversity and are representative of ecosystems. |
| Global Biodiversity Information Facility* | GBIF | <http://www.gbif.org/> | Houses over 1.6 billion species occurrence records from over 54,600 data sets (as of October 2020). |
| Global Environmental Flow Information System* | International Water Management Institute | <http://gef.iwmi.org/> | Interactive maps provide information related to Environmental Flows for current conditions and different Environmental Management Classes of a river system based on river health indicators. |
| Global Marine Environment Datasets – GMED* | GEOBON | <http://gmed.auckland.ac.nz/index.html> | Climatic, biological and geophysical environmental layers of both present day, past and future environmental conditions. For use with species distribution modelling software like Maximum entropy (MaxENT) and for any other marine environment visualisation exercise. |
| Global Forest Change* | University of Maryland | <https://earthenginepartners.appspot.com/science-2013-global-forest> | Results from time-series analysis of Landsat images characterizing forest extent and change. Also of use in monitoring forest loss. |
| Global Forest Watch* (including GFW Climate, GFW Commodities, GFW Fires) | World Resources Institute | <http://www.globalforestwatch.org/> | Near-real-time data and tools for monitoring forests. Offers weekly GLAD deforestation alerts that show where tree cover loss is happening in the tropics. Includes several related tools: GFW Climate (data on forest carbon); GFW Commodities (data on forest change, concessions, mills, etc to assess supply chain risks); GFW Fires (near real-time information on forest and land fires). |
| Global Mangrove Watch* | Aberystwyth University (U.K.), solo Earth Observation (soloEO; Japan), Wetlands International, UNEP-WCMC, Japan Aerospace Exploration Agency (JAXA). | <https://www.globalmangrovewatch.org/>  <https://gma-panda.opendata.arcgis.com/> | Data and maps on mangrove extent and biomass in the tropics and subtropics (contributing to Ramsar Global Wetlands Observing System). |
| iNaturalist* | California Academy of Sciences and National Geographic Society | <https://www.inaturalist.org/> | Citizen science nature observations. |
| International Waterbird Census Database* | Wetlands International | <http://wpe.wetlands.org/> | Current and historic estimates, trends and 1% thresholds for over 800 waterbird species and 2,300 biogeographic populations worldwide. |
| IUCN Red List of Ecosystems* | IUCN | <https://iucnrle.org/assessments/> | Status of a number of ecosystems. |
| IUCN Red List of Threatened Species* | The Red List Partnership – 10 organisations led by IUCN | <https://www.iucnredlist.org/> | Extinction risk of species with data on range, population trends, habitat use, life history traits, use and trade, threats, conservation actions currently in place and conservation actions needed. |
| Living Planet Index* | WWF, Zoological Society of London | <http://www.livingplanetindex.org/data_portal> | Trends in over 27,000 populations of more than 4,700 vertebrate species. |
| Map of Life* | Yale University, University of Florida | <https://mol.org/datasets/> | Accesses data on plants and animals from multiple sources (local inventories, regional checklists, expert range maps such as IUCN Red List, gridded surveys, point observations and Wikipedia). |
| Movebank* | Max Planck Institute for Ornithology | <https://www.movebank.org/> | Animal tracking data. |
|  |  |  |  |
| Ocean Health Index* | National Center for Ecological Analysis and Synthesis (NCEAS) and Conservation International | <http://ohi-science.org/>  <http://www.oceanhealthindex.org/region-scores/annual-scores-and-rankings> | Annual national scores for biological, physical, economic, and social aspects of ocean health since 2014. |
| Ocean Tracking Network Data Portal* | Dalhousie University, Canada | <http://members.oceantrack.org/> | Data from the tracking of aquatic animals |
| PlantLife Important Plant Areas Database* | PlantLife International | <http://www.plantlifeipa.org/home> | Data on Important Plant Areas, including species, habitats, IPA criteria, threats, land use, and protection, as well as textual accounts of the sites. |
| SWOT – The State of the World’s Sea Turtles* | Partnership including Oceanic Society, IUCN SSC Marine Turtle Specialist Group, Duke University's OBIS-SEAMAP, and a growing network of institutions and individuals. | <https://www.seaturtlestatus.org/online-map-data> | Data collated on marine turtles e.g. nesting data, satellite telemetry data, species distributions, Regional Management Unit boundaries, genetic data, etc. No time-series population data. |
| ThreatSearch* | Botanic Gardens Conservation International | <https://tools.bgci.org/threat_search.php> | Database of conservation assessments of plants. |
| Water-related Ecosystems* | UN Environment | <https://www.sdg661.app/> | National, sub-national, basin and sub-basin aggregated data on water extent. |
| Water Quality Index for Biodiversity | United Nation’s Environment Programme’s Global Environment Monitoring System for Water (GEMS/Water) | <https://www.bipindicators.net/indicators/water-quality-index-for-biodiversity>  <https://www.unep.org/explore-topics/water/what-we-do/monitoring-water-quality> | Data on water quality over time, apparently collated by UNEP from national reports. |
| Wetland Extent Trends (WET) Index database | UNEP-WCMC | <https://www.bipindicators.net/indicators/wetland-extent-trends-index>  <https://www.sciencedirect.com/science/article/abs/pii/S0006320715301476> | Time-series data (available on request) on change in area of natural wetland |
| Wildlife Insights database* | Wildlife Insights/CI | <https://wildlifeinsights.org/team-network>  <https://app.wildlifeinsights.org/explore> | Camera trap data on nearly 1,000 species. |
| World Database on Key Biodiversity Areas | Managed by BirdLife International for the KBA Partnership | <http://www.keybiodiversityareas.org/home> | Information on KBAs (available on request for non-commercial purposes). |
| Zoological Information Management System (ZIMS) | Species 360 | <https://www.species360.org/products-services/zims-for-husbandry/> | Data (available to members) on animal accessions and dispositions, animals wanted and available, behavioural observations, feed logs, etc. Generates information needed for CITES and other permitting uses. Data on generation time important for IUCN Red List assessments. |
