# Supplementary material for: An inventory of biodiversity data sources for conservation monitoring
Source: PLoS One. 2020 Dec 2;15(12):e0242923. doi: 10.1371/journal.pone.0242923 (PMC7710106; doi:10.1371/journal.pone.0242923)
Supplement: S2 Table — Those data sources where at least some data (or mapping of data) appear to be instantly, freely and openly available are flagged with a star (*). Note that some data sources would need updating before they could be of use. An updated list, with additional information, will be posted on https://www.speciesmonitoring.org/data-sources.html. Data source managers are encouraged to send any additional information or updates to SpeciesMonitoringSG@gmail.com. (DOCX) [file pone.0242923.s002.docx]

**S2 Table.** **Global data sources of potential value in monitoring pressures and threats to biodiversity.** Those data sources where at least some data (or mapping of data) appear to be instantly, freely and openly available are flagged with a star (*). Note that some data sources would need updating before they could be of use. An updated list, with additional information, will be posted on https://www.speciesmonitoring.org/data-sources.html. Data source managers are encouraged to send any additional information or updates to SpeciesMonitoringSG@gmail.com.

| **Data source** | **Lead agency** | **URL** | **Description** |
| --- | --- | --- | --- |
| **Multiple threats** | | |  |
| Cumulative Human Impacts on Marine Ecosystems* | Halpern lab | <https://www.nature.com/articles/ncomms8615#supplementary-information> | Recent change over 5 years in cumulative impacts to marine ecosystems globally from fishing, climate change, and ocean- and land-based stressors. |
| Forest Atlases* | WRI | <https://www.wri.org/our-work/project/forest-atlases> | Online information systems to visualize and analyse data on forest change, land use, and land cover. |
| Global Database on Sustainable Land Management* | United Nations Convention to Combat Desertification (UNCCD) | <https://www.wocat.net/en/global-slm-database> | Data to assess spatial coverage of land degradation and sustainable land management (the use of soil, water, vegetation and animals to produce goods and services) |
| Global Footprint Network’s National Footprint Accounts* | Global Footprint Network | <http://data.footprintnetwork.org/?_ga=2.22111038.422986948.1602489816-449568750.1602489816#/> | Time-series data on the gap between human demand on nature (Ecological Footprint) and nature’s capacity to meet that demand (biological capacity) for over 200 countries and regions from 1961. |
| History Database of the Global Environment (HYDE)* | PBL Netherlands Environmental Assessment Agency | <https://themasites.pbl.nl/tridion/en/themasites/hyde/download/index-2.html> | Data on human populations and land use change going back centuries. |
| Land Use Harmonization V2* | Global Ecology Laboratory, University of Maryland | <https://luh.umd.edu/data.shtml> | Modelled land use states for the globe from year 800 AD. Land use categories include: primary and secondary natural vegetation in forest and non‐forest subtypes, pasture, and croplands. |
| PREDICTS Database  (Projecting Responses of Ecological Diversity In Changing Terrestrial Systems)* | UNEP-WCMC, Natural History Museum (London) | <https://www.predicts.org.uk/>  <https://data.nhm.ac.uk/dataset/the-2016-release-of-the-predicts-database> | Global database of terrestrial species' responses to human pressures collated from existing spatial comparisons of local-scale biodiversity exposed to different intensities and types of pressures |
| Resilience Atlas* | Conservation International | <https://www.resilienceatlas.org/> | Tool for understanding the extent/severity of stressors affecting rural livelihoods, production systems, and ecosystems and the ways different wealth and assets impact resilience. Includes data on pressures such as forest loss and pollution. |
| Several data sources relate to habitat loss which can be caused by many (arguably all) of the threats listed below. In addition, many of the databases for monitoring the state of habitats (Table S1) can also be used to monitor habitat loss. | | | |
| **Residential & Commercial Development** | | |  |
| *Anthropogenic biomes in SEDAC* (see S4 Table)* | *NASA's Earth Observing System Data and Information System (*EOSDIS*) hosted by* CIESIN *at Columbia University* | <https://sedac.ciesin.columbia.edu/data/collection/anthromes/sets/browse>  *https://sedac.ciesin.columbia.edu/data/sets/browse?title=urban+extent* | *Data on anthropogenic biomes and urban extent (Data source assessed in S4 Table).* |
| **Agriculture & Aquaculture** | | |  |
| Forest 500* | Global Canopy | <https://forest500.org/data/companies> | Ranks annually the most influential companies and financial institutions in forest risk commodity supply chains. |
| Global data on forest plantations resources* | FAO | <http://www.fao.org/3/Y2316E/y2316e0b.htm> | Area under plantation and species used. Needs updating. |
| OECD Agriculture Statistics* | OECD | <https://www.oecd-ilibrary.org/agriculture-and-food/data/oecd-agriculture-statistics_agr-data-en> | Agriculture data for OECD countries and non-members, including forecasts on the evolution of agricultural markets and commodities, estimates of policy support, and indicators of environmental performance of agriculture. |
| Trase* | Stockholm Environment Institute, Global Canopy  with Vizzuality and the European Forest Institute | <https://trase.earth/about> | Maps the links between consumer countries via trading companies to the places of production, showing how commodity exports are linked to agricultural conditions |
| **Transportation & Service Corridors** | | |  |
| Global Roadkill Network Data | Global Roadkill Network | <http://globalroadkill.net/programs/progress.html> | National data on road kill. Under development; not yet ready for use. |
| IWC Global Ship Strikes Database | International Whaling Commission | <https://iwc.int/ship-strikes> | Information on reported ship strikes, both historic and current. |
| **Biological Resource Use** | | |  |
| Bushmeat Database | CIFOR | <https://www.cifor.org/bushmeat/resources/bushmeat-database/>  <https://www2.cifor.org/bushmeat/resources/bushmeat-data-map/> | A repository of papers and reports providing access to data on an important driver of species loss. |
| CITES Trade Database* | Maintained by UNEP-WCMC on behalf of the CITES Secretariat | <https://trade.cites.org/> | Massive database showing trade records by country. Can also be used to assess conservation actions relating to trade. |
| *Related databases:*  *Big Cat Trade Dashboard**  *ETIS – Elephant Trade Information System** | *UNEP-WCMC*  *CITES* | <https://app.powerbi.com/view?r=eyJrIjoiYzU1ZjZkOGYtZTU0OC00YzNhLThlZjMtNmM3YzU2N2ZkYzgzIiwidCI6IjJmYWFiODU4LWQxZjQtNDhhZi04NmY2LTQ4NjE5NmQ1OTY5ZCIsImMiOjh9>  <https://www.cites.org/eng/prog/etis/index.php> | *Data for big cat species in dashboard format. If this is going to be updated regularly, it could be of interest in visualizing trade trends for key species.*  *Information system to track illegal trade in ivory and other elephant products around the world.* |
| Estimates of Global Fishery Bycatch and Discards* | FAO | <http://www.fao.org/3/t4890e/T4890E02.htm> | Qualitative and quantitative data on the character and magnitude of discards occurring in various fisheries of the world. |
| Fisheries and Aquaculture database* | FAO | <http://www.fao.org/fishery/statistics/collections/en> | Multiple fisheries datasets. The ones relevant to fishing and monitoring overfishing include: Global Production, Global Capture Production, Global Tuna Catches by Stock, Global Aquaculture Production, Atlas of Tuna and Billfish Catches, Global Number of Fishers, Fishery Commodities and Trade, Consumption of Fish and Fishery Products. |
| Global Fishing Watch* | Oceana, SkyTruth,  Google | <https://globalfishingwatch.org/map-and-data/> | Tool to visualise, track and share data on global fishing activity in near real-time. |
| ICES Datasets* | International Council for the Exploration of the Sea | <https://www.ices.dk/data/dataset-collections/Pages/default.aspx> | Datasets on the marine environment, including fish stocks and catches. |
| Marine Trophic Index* | Sea Around Us (University of British Columbia) | <http://www.seaaroundus.org/data/#/marine-trophic-index> | The mean trophic level for all Large Marine Ecosystems - a measure of whether fish stocks are being overexploited or sustainably managed. |
| The ROUTES Dashboard* | USAID  ROUTES Partnership including the Center for Advanced Defense Studies (C4ADS), Freeland, International AirTransport Association (IATA), TRAFFIC and WWF. | <http://www.routesdashboard.org/> | Graphics on wildlife trafficking through airports. |
| Vulnerable Marine Ecosystems Database* | FAO | <http://www.fao.org/in-action/vulnerable-marine-ecosystems/vme-database/en/vme.html> | Global inventory of fisheries measures to protect vulnerable marine ecosystems in areas beyond national jurisdictions. |
| **Natural System Modifications** | | |  |
| Global Dam Watch* | Global Dam Watch | <http://globaldamwatch.org/> | Portal to data from three databases on dams: GlObal geOreferenced Database of Dams (GOOD2), Global Reservoir and Dam Database (GRanD), and Future Hydropower Reservoirs and Dams (FHReD). |
| **Invasive & Problematic Species, Pathogens & Genes** | | |  |
| Global Register of Introduced and Invasive Species (GRIIS) | Led by the IUCN SSC Invasive Species Specialist Group for the Global Invasive Alien Species Information Partnership. | <http://www.griis.org/about.php> | Annotated and verified national inventories of introduced and invasive species (under development). |
| Invasive Species Compendium* | CAB International | <https://www.cabi.org/ISC/> | Range of science-based information to support decision-making in invasive species management worldwide. |
| IUCN Global Invasive Species Database (GISD)* | IUCN SSC Invasive Species Specialist Group | <http://www.iucngisd.org/gisd/> | Data on alien and invasive species that negatively impact biodiversity. |
| Threatened Island Biodiversity (TIB) Database* | TIB partnership: Island Conservation, University of California at Santa Cruz, BirdLife International, IUCN SSC Invasive Species Specialist Group. | <http://tib.islandconservation.org/> | Global database of threatened island species at risk from invasive vertebrates. |
| **Pollution** | | |  |
| Global Health Observatory Data Repository* | World Health Organisation | <http://apps.who.int/gho/data/node.main.152?lang=en> | Includes data on exposure to ambient air pollution, which will be just as relevant to wildlife as people. Needs updating. |
| Global Pollution Map* | Global Alliance on Health and Pollution | <https://www.pollution.org/> | Data on air pollution and contaminated sites. |
| Litterbase* | Alfred Wegener Institute, Helmholtz Centre for Polar and Marine Research | <https://litterbase.awi.de/> | Results from over 2,000 scientific studies on marine litter in global maps and figures. Includes data on the amount and distribution of litter and microplastics, and Interactions between aquatic life and marine litter. |
| Loss of Reactive Nitrogen to the Environment | York University | <https://www.bipindicators.net/indicators/trends-in-loss-of-reactive-nitrogen-to-the-environment> | The reactive nitrogen loss for different countries as a result of the production and consumption of food and the use of energy; data need updating and need to be requested. |
| Nitrogen Deposition Indicator | York University | <https://www.bipindicators.net/indicators/trends-in-nitrogen-deposition> | Shows where energy and food production have resulted in increased ammonia and nitrogen oxide emissions, with subsequent increase in nitrogen depositions. Data need updating and need to be requested. |
| **Climate change** | | |  |
| Carbon Disclosure Project Data Portal* | CDP | <https://data.cdp.net/> | Carbon-related data for cities and regions including greenhouse gas (GHG) emissions, renewable energy, water resources, etc. |
| Climate Change Indicators* | US Environmental Protection Agency | <https://www.epa.gov/climate-indicators> | A diversity of climate-related data including GHG emissions, temperatures, bird wintering ranges, sea surface temperatures, etc. |
| Climate Change Initiative* | European Space Agency | <https://www.esa-landcover-cci.org/?q=node/158> | Maps on land cover, land use change and water bodies. |
| Climate Watch* | WRI | <https://www.climatewatchdata.org/> | Data on GHG emissions and countries’ Nationally Determined Contributions. |
| Coral Reef Watch* | NOAA – US National Oceanic and Atmospheric Administration | <https://coralreefwatch.noaa.gov/satellite/index.php> | Satellite data to provide current reef environmental conditions to quickly identify areas at risk for coral bleaching. Includes Daily Global 5km Satellite Coral Bleaching Heat Stress Alert Area (real-time monitoring of coral bleaching hear stress) |
| GHG Data from UNFCCC* | UNFCCC | <https://unfccc.int/process/transparency-and-reporting/greenhouse-gas-data/ghg-data-unfccc> | GHG emissions by country |
| Global Climate Change* | NASA | <https://climate.nasa.gov/vital-signs/carbon-dioxide/> | Data on carbon dioxide levels, global temperatures, Arctic sea ice minimum, ice sheets and sea level. |
| Notre Dame Global Adaptation Index (ND-GAIN)* | University of Notre Dame Global Adaptation Initiative | <https://gain.nd.edu/our-work/country-index/> | An annual index of climate change adaptation composed of a vulnerability score and a readiness score. |
| OECD Inventory of Support Measures  for Fossil Fuels* | OECD - Organisation for Economic Co-operation and Development | <http://www.oecd.org/fossil-fuels/data/> | Online inventory of support measures for  fossil fuels, which identifies, documents, and estimates the value of support arising from more than 1,000 individual policies that encourage the production or consumption of fossil fuels. |
| Water Information Network System - IHP-WINS* | UNESCO | <http://ihp-wins.unesco.org/> | Water-related information ranging from groundwater to urban water through gender issues, from local to global scale. Includes data on level of water stress (SDG Indicator 6.4.2) and Environmental Water Stress in Transboundary River Basins. |
| World Weather and Climate Extremes Archive* | Arizona State University for the World Meteorological Organisation | <https://wmo.asu.edu/#global> | Data on world weather extremes. |
