# Supplementary material for: An inventory of biodiversity data sources for conservation monitoring
Source: PLoS One. 2020 Dec 2;15(12):e0242923. doi: 10.1371/journal.pone.0242923 (PMC7710106; doi:10.1371/journal.pone.0242923)
Supplement: S3 Table — Those data sources where at least some data appear to be freely and openly available are flagged with a star (*). Note that some data sources would need updating before they could be of use. An updated list, with additional information, will be posted on https://www.speciesmonitoring.org/data-sources.html. Data source managers are encouraged to send any additional information or updates to SpeciesMonitoringSG@gmail.com. (DOCX) [file pone.0242923.s003.docx]

**S3 Table. Global data sources of potential value in monitoring conservation responses to biodiversity loss.** Those data sources where at least some data appear to be freely and openly available are flagged with a star (*). Note that some data sources would need updating before they could be of use. An updated list, with additional information, will be posted on https://www.speciesmonitoring.org/data-sources.html. Data source managers are encouraged to send any additional information or updates to SpeciesMonitoringSG@gmail.com.

| **Data source** | **Lead agency** | **URL** | **Description** |
| --- | --- | --- | --- |
| **Land/Water Management** | | |  |
| Atlas of Forest Landscape Restoration Opportunities* | World Resources Institute in collaboration with the University of Maryland and IUCN (as a contribution to the Global Partnership on Forest Landscape Restoration) | <https://www.wri.org/our-work/project/global-restoration-initiative/methodology-about-atlas-forest-landscape-restoration> | Global approximation of where degraded forest lands have the potential to be restored. Includes maps on current forest cover, potential forest cover, forest condition, restoration opportunities and human pressure. |
| Restoration Evidence* | Cambridge Conservation Initiative Endangered Landscape Programme | <http://www.restorationevidence.org/> | Evidence for the effectiveness of ecological restoration management actions. Contains summaries of scientific research on the effects of actions to restore habitats, categorized by the target habitat or species |
| The Bonn Challenge Barometer* | IUCN and partners | <https://infoflr.org/bonn-challenge-barometer> | Action on Bonn Challenge commitments for Forest Landscape Restoration, including data on success factors, and results & benefits. Limited global coverage so far. |
| **Species Management** | | |  |
| Database of Island Invasive Species Eradications* | Island Conservation, University of California at Santa Cruz, IUCN SSC Invasive Species Specialist Group, University of Auckland, Landcare Research New Zealand | <http://diise.islandconservation.org/> | Data from historical and current invasive vertebrate eradication projects on islands (mostly focused on invasive mammals) including: island location and characteristics, details about the eradication (focal species, methods, outcome, etc), and links and contacts. |
| PlantSearch* | Botanic Gardens Conservation International | <https://tools.bgci.org/plant_search.php> | Global database of living plant, seed and tissue collections. |
| Zoo and Aquarium Statistics* | Association of Zoos & Aquariums | <https://www.aza.org/zoo-and-aquarium-statistics> | Data on species held in captivity, including populations, life expectancy, etc. |
| **Livelihood, Economic & Moral Incentives** | | |  |
| Area of forest under sustainable management through FSC* | Forest Stewardship Council | <https://ic.fsc.org/en/facts-and-figures> | Figures on area of forest under FSC certified production. |
| Area of forest under sustainable management through PEFC* | Programme for the Endorsement of Forest Certification | <https://www.pefc.org/discover-pefc/facts-and-figures> | Figures on area of forest under PEFC certified production. |
| ASC Certification Updates* | Aquaculture Stewardship Council | <https://www.asc-aqua.org/news/certification-update/> | Monthly update on the progress of the ASC programme (number of sites, production volume, etc). |
| BCI Key Facts* | Better Cotton Initiative | <https://bettercotton.org/resources/key-facts/> | Data on numbers of farms under sustainable cotton production, tonnes and hectares produced, etc. |
| Gini Index* | World Bank | <https://data.worldbank.org/indicator/SI.POV.GINI>  <https://resourcewatch.org/data/explore/GINI-Index> | The extent to which the distribution of income within an economy deviates from a perfectly equal distribution. |
| Human Development Index* | UNDP | <http://hdr.undp.org/en/content/human-development-index-hdi>  <http://hdr.undp.org/en/data> | Average achievement in dimensions of human development: long and healthy life, being knowledgeable; having a decent standard of living. |
| Multidimensional Poverty Index* | UNDP | <http://hdr.undp.org/en/2018-MPI> | Deprivations at the household and individual level in health, education, and standard of living. |
| RSPO Certification* | Roundtable on Sustainable Palm Oil | <https://rspo.org/impacts> | Data on the area of land under RSPO certified production. |
| RTRS Certified Volumes and Producers* | Round Table for Responsible Soy Association | <https://responsiblesoy.org/?lang=en> | Data on the production of soy that follows the RTRS Standard for Responsible Soy Production. Also calculates soy footprint. |
| MSC Certified Catch* | Marine Stewardship Council | <https://www.msc.org/what-we-are-doing/our-collective-impact> | Data on proportion of fisheries under MSC and details of specific fisheries. |
| Proportion of fish stocks within biologically sustainable levels* | FAO | <http://www.fao.org/sustainable-development-goals/indicators/1441/en/> | Time-series data presented on proportion of fish stocks within biologically sustainable levels. |
| In addition to the data sets mentioned, other organisation involved in the sustainable production of commodities also provide data on volumes of certified crops produced and the hectares under sustainable management. Examples include:   - Bonsucro (sugar) - Rainforest Alliance (cattle, cocoa, coffee, cut flowers, palm oil, tea) - Roundtable on Sustainable Biomaterials (bio-based feedstock, biomass-derived material and any advanced fuel or product) - UTZ (cocoa, coffee hazelnuts, tea). | | |  |
| **Conservation Designation & Planning** | | |  |
| DOPA - the Digital Observatory for Protected Areas* | JRC | <https://dopa.jrc.ec.europa.eu/en/mapsanddatasets>  <https://dopa-explorer.jrc.ec.europa.eu/> | As well as PA details extracted from Protected Planet, DOPA Explorer has tabs showing IUCN Red List species whose range maps overlap with the protected area. |
| Global Database on Protected Area Management Effectiveness (GD-PAME)* | UNEP-WCMC | <https://pame.protectedplanet.net/> | Results of management effectiveness assessments for protected areas. |
| MPAtlas* | Marine Conservation Institute | <http://www.mpatlas.org/> | Data on level of protection and implementation of the world's marine protected areas. |
| Protected Planet – World Database on Protected Areas* | Lead: UNEP-WCMC with IUCN and UN Environment | <https://www.protectedplanet.net/> | Data on the world’s protected areas. Now also includes data on Other Effective Area-Based Conservation Measures. |
| Protected Area Connectedness Index (PARC-Connectedness) | CSIRO, GEO BON, GBIF, Map of Life | <https://www.bipindicators.net/indicators/protected-area-connectedness-index-parc-connectedness> | Data on the extent to which terrestrial protected areas form “well-connected systems of protected areas … integrated into the wider landscape”. Need to ask for data. |
| Protected Area Representativeness Index (PARC-Representativeness) | CSIRO, GEO BON, GBIF, Map of Life | <https://www.bipindicators.net/indicators/protected-area-representativeness-index-parc-representativeness> | Data on the extent to which terrestrial protected areas are “ecologically representative”. Need to ask for data. |
| Ramsar Sites Information service* | Ramsar Secretariat | <https://rsis.ramsar.org/?pagetab=2> | Data on number of Ramsar sites and area covered. |
| **Legal & Policy Frameworks** | | |  |
| Climate Change Laws of the World Database* | London School of Economics | <http://www.lse.ac.uk/GranthamInstitute/climate-change-laws-of-the-world/> | Database of climate and climate-related laws, as well as laws and policies promoting low carbon transitions. |
| Policy Instruments for the Environment (PINE) Database | OECD - Organisation for Economic Co-operation and Development | <https://www.oecd.org/environment/indicators-modelling-outlooks/policy-instrument-database/> | Information on policy instruments relevant for environmental protection and natural resource management. |
| Species+* | UNEP-WCMC, CITES Secretariat | <https://www.speciesplus.net/species> | Data on species currently listed in the Appendices of CITES, CMS and other conventions (taxonomy, distribution, trade restrictions, etc). |
| The Policy Climate Interactive* | Climate Policy Initiative | <http://www.thepolicyclimate.org/> | Policy issues in key countries relevant to climate change with evidence on emissions trends, emissions drivers, and policy activity. |
| **Research and Monitoring** | | |  |
| UIS Database* | UNESCO Institute of Statistics | <http://data.uis.unesco.org/> | Includes data on funds invested in research and development. |
| **Institutional Development (including financing)** | | |  |
| Classification of Functions of the Government (COFOG) | OECD - Organisation for Economic Co-operation and Development | <https://datahub.io/core/cofog#data>  <https://www.oecd-ilibrary.org/governance/government-at-a-glance-2017/classification-of-the-functions-of-government-cofog_gov_glance-2017-94-en> | Includes data on biodiversity expenditure. |
| Financing for Sustainable Development* | OECD | <http://www.oecd.org/dac/financing-sustainable-development/> | Data on official and private resource flows. |
| FundingTheOcean. Org | Foundation Maps | <https://maps.foundationcenter.org/#/map/> | Ocean conservation funding. Data for sale. |
| World Carbon Market Database* | Carbon Market Data | <https://www.carbonmarketdata.com/en/products/world-ets-database/presentation> | Includes information on carbon trading schemes. |
