# Supplementary material for: An inventory of biodiversity data sources for conservation monitoring
Source: PLoS One. 2020 Dec 2;15(12):e0242923. doi: 10.1371/journal.pone.0242923 (PMC7710106; doi:10.1371/journal.pone.0242923)
Supplement: S4 Table — Those data sources where at least some data seem to be freely and openly available are flagged with a star (*). Note that some data sources would need updating before they could be of use. An updated list, with additional information, will be posted on https://www.speciesmonitoring.org/data-sources.html. Data source managers are encouraged to send any additional information or updates to SpeciesMonitoringSG@gmail.com. (DOCX) [file pone.0242923.s004.docx]

**S4 Table.** **Global data sources with multiple uses for biodiversity monitoring**. Those data sources where at least some data seem to be freely and openly available are flagged with a star (*). Note that some data sources would need updating before they could be of use. An updated list, with additional information, will be posted on https://www.speciesmonitoring.org/data-sources.html. Data source managers are encouraged to send any additional information or updates to SpeciesMonitoringSG@gmail.com.

| **Data source** | **Lead agency** | **URL** | **Description** |
| --- | --- | --- | --- |
| Aquastat* | FAO | <http://www.fao.org/nr/water/aquastat/data/query/index.html?lang=en> | Huge dataset on water resources and water use by country. |
| Aqueduct* | WRI | <https://www.wri.org/our-work/project/aqueduct> | Data on water risks. |
| Data Basin* | Conservation Biology Institute | <https://databasin.org/> | A mapping and analysis platform linked to over 20,000 environmental datasets. |
| DataONE* | Data Observation Network for Earth | <https://www.dataone.org/> | Access to over 820,000 Earth and environmental data sets across multiple repositories (including citizen science data). |
| Earth Pulse and Vital Signs of the Planet | National Geographic Society Geographic Visualization Lab | <https://www.nationalgeographic.org/projects/labs/geographic-visualization/> | Platform under development to monitor and visualize data on biodiversity, migration, extreme environments, critical ecosystems, human pressures, and protected areas. |
| Environmental Performance Index* | Yale University, Columbia University | <https://epi.envirocenter.yale.edu/> | Every 2 years, ranks countries on 24 performance indicators across 10 issue categories (air quality, water quality, heavy metals, biodiversity, forests, fisheries, climate & energy, air pollution, water resources, agriculture). |
| EOSDIS Earthdata* | NASA | <https://earthdata.nasa.gov/> | Raw land and atmosphere data for use by GIS specialists. Includes daily updates on fires. |
| Environment Live* (rebranded as World Environment Situation Room) | UN Environment | <http://uneplive.unep.org/> | Mostly human welfare related data and pollution-related data. Potentially useful time-series data include ocean pollution (shipping lanes, ports) and commercial activity (shipping lanes), as well as biodiversity state data from Protected Planet, IUCN Red List and Ramsar. |
| Environmental Data Explorer* | UN Environment | <http://geodata.grid.unep.ch/> | Data on more than 500 variables used to produce Global Environment Outlooks, on themes like Freshwater, Population, Forests, Emissions, Climate, Disasters, Health and GDP. Needs updating. |
| Environmental Sustainability Index* | Yale Center for Environmental Law and Policy, Center for International Earth Science Information Network at Columbia University, World Economic Forum | <https://ec.europa.eu/jrc/en/publication/articles-books/environmental-sustainability-index-esi> | The ESI integrates 76 variables into 21 indicators of environmental sustainability for 146 countries in 5 categories: environmental systems, reducing environmental stresses, reducing human vulnerability to environmental stresses, societal and institutional capacity to respond to environmental challenges, and global stewardship. Needs updating. |
| FAOSTAT* | FAO | <http://www.fao.org/faostat/en/#data> | Huge database with several useful environmental datasets. Includes Land Use which includes area of land under primary forest, agriculture, fisheries, livestock, etc. Some data out of date. |
| GEOBON Essential Biodiversity Variables Data Portal* | GEOBON | https://portal.geobon.org/ | EBV data including changes in: forest cover (from Landsat), species diversity (from PREDICTS) and bird diversity (using countryside species-area relationship models from PREDICTS). |
| The Global Earth Observation System of Systems (GEOSS) Portal* | GEOSS | <http://www.geoportal.org/> | Various data overlays, including habitat types and threatened species. |
| GloVis* | US Geological Survey | <https://glovis.usgs.gov/> | Visualization of satellite-based remote sensing datasets. |
| IBAT - Integrated Biodiversity Assessment Tool* | IBAT Alliance: BirdLife International, CI, IUCN, UNEP-WCMC | <https://www.ibat-alliance.org/> | Platform to access and analyse data from IUCN Red List of Threatened Species, World Database on Protected Areas and World Database of Key Biodiversity Areas. |
| Map X* | University of Geneva, UNEP-GRID, UNEP | <https://www.mapx.org/> | An online platform for managing geospatial data on natural resources and the environment. |
| Mountain Portal* | Global Mountain Biodiversity Assessment | <http://www.mountainbiodiversity.org/explore> | Data on mountain biodiversity inked to Map of Life. Includes data on species ranges from various sources (including IUCN Red List, eBird, etc). |
| NatureServe Dashboard* | NatureServe | <http://dashboard.natureserve.org/> | Dashboard that presents data derived from multiple sources on biodiversity states (e.g. forest cover, water quality, genetic diversity of livestock) pressures (e.g. human footprint, forest cover loss) responses (e.g. ASEAN Heritage parks, GBIF records) and benefits (carbon sequestration, freshwater provision). |
| Ocean Action Hub* | UNDP | <https://www.oceanactionhub.org/sdg-14-targets-context-and-indicators> | Data on each of the SDG 14 Life Below Water indicators. Also has list of other relevant marine databases. |
| Ocean Biogeographic Information System – OBIS* | Intergovernmental Oceanographic Commission of UNESCO | <https://obis.org/> | Huge global database on marine species linked to GBIF. Over 164 million records of over 137,000 species from more than 3,300 datasets (as of October 2020). |
| OBIS-SEAMAP* | Duke University | <http://seamap.env.duke.edu/> | Spatially referenced database aggregating marine mammal, seabird, sea turtle and ray & shark observation data. |
| Ocean Data Viewer* | UNEP-WCMC | <http://data.unep-wcmc.org> | Includes data on global patterns and predictors of marine biodiversity across taxa and several species richness and cetacean distribution maps. |
| Ocean+ Library* | UNEP-WCMC | <https://library.oceanplus.org/> | An overview of global marine and coastal datasets of biodiversity importance. |
| PANGAEA* | Alfred Wegener Institute, Helmholtz Center for Polar and Marine Research, and the Center for Marine Environmental Sciences, University of Bremen | <https://www.pangaea.de/> | A data publisher for earth & environmental science. Large diversity of data sets available. |
| Reefbase – A Global Information System for Coral Reefs* | ReefBase | <http://www.reefbase.org/global_database/default.aspx> | Country-level data on coral reef resources, status, threats and management. Needs updating. |
| Resource Watch* | WRI and 35 partners | <https://resourcewatch.org/> | Data visualization platform under development with over 300 data sets on topics ranging from climate change to human migration, deforestation to air quality, agriculture to energy and more. Links to several biodiversity-related databases such as Protected Planet. |
| SDG Indicators Metadata Repository* | UN Statistics Division | <https://unstats.un.org/sdgs/metadata/> | Data and statistics for the Tier I and II indicators in the global indicator framework for the SDGs. |
| Socioeconomic Data and Applications Center (SEDAC)* | NASA's Earth Observing System Data and Information System (EOSDIS) hosted by CIESIN at Columbia University | <http://sedac.ciesin.columbia.edu/theme/conservation/data/sets/browse> | Includes a large diversity of data sets (some out of date), including anthropogenic biomes of the world, global species richness for mammals and amphibians, Global Human Influence Index, Human footprint and Environmental Performance Index. |
| UN Biodiversity Lab* | UNDP, UN Environment | <https://www.unbiodiversitylab.org/index.html> | Online platform to access global datasets relating to the Aichi Biodiversity Targets and nature-based Sustainable Development Goals. |
