# Supplementary material for: An inventory of biodiversity data sources for conservation monitoring
Source: PLoS One. 2020 Dec 2;15(12):e0242923. doi: 10.1371/journal.pone.0242923 (PMC7710106; doi:10.1371/journal.pone.0242923)
Supplement: S5 Table — (DOCX) [file pone.0242923.s005.docx]

**S5 Table. Reports synthesising large biodiversity data sets over time.**

| **Report** | **Lead agency** | **URL** |
| --- | --- | --- |
| Atlas of Sustainable Development Goals | World Bank | <https://blogs.worldbank.org/opendata/2018-atlas-sustainable-development-goals-all-new-visual-guide-data-and-development> |
| EarthPulse | National Geographic Society | <https://www.amazon.co.uk/National-Geographic-EarthPulse-Society-2010-10-18/dp/B01FIWBH6A/ref=sr_1_13?keywords=earthpulse&qid=1558797125&s=books&sr=1-13> |
| Ecological Footprint Report | Ecological Footprint Network | <https://www.footprintnetwork.org/resources/publications/> |
| Environmental Outlook | OECD - Organisation for Economic Co-operation and Development | <http://www.oecd.org/environment/indicators-modelling-outlooks/outlooks.htm> |
| Fishery and Aquaculture Statistics | FAO | <http://www.fao.org/fishery/statistics/programme/publications/all/en> |
| Global Assessment of Animal Genetic Resources | FAO | <http://www.fao.org/publications/sowangr/en/> |
| Global Biodiversity Outlook | Secretariat of the Convention on Biological Diversity | <https://www.cbd.int/gbo/> |
| Global Environment Outlook | UN Environment | <https://www.unenvironment.org/global-environment-outlook> |
| Global Wetland Outlook: State of the World’s Wetlands and Their Services to People | Secretariat of the Ramsar Convention | <https://www.ramsar.org/sites/default/files/flipbooks/ramsar_gwo_english_web.pdf> |
| IPBES Global Assessment Reports including:  Land Degradation and Restoration  Pollinators, Pollination and Food Production  Scenarios and Models of Biodiversity and Ecosystem Services. | The Intergovernmental Science-Policy Platform on Biodiversity and Ecosystem Services - IPBES | <https://www.ipbes.net/assessment-reports> |
| Living Planet Report | WWF, Zoological Society of London | <https://livingplanet.panda.org/en-us/> |
| OECD-FAO Agricultural Outlook | OECD, FAO | <https://www.oecd-ilibrary.org/agriculture-and-food/oecd-fao-agricultural-outlook-2020-2029_1112c23b-en> |
| Protected Planet Report | UNEP-WCMC, IUCN, National Geographic Society | <https://www.protectedplanet.net/en/resources/global-reports> |
| Renewables Global Status Report | REN21 | <http://www.ren21.net/status-of-renewables/global-status-report/> |
| The State of the World’s Biodiversity for Food and Agriculture | FAO | <http://www.fao.org/3/CA3129EN/CA3129EN.pdf> |
| State of the World’s Birds | BirdLife International | http://datazone.birdlife.org/2020-annual-update |
| State of the World’s Fisheries and Aquaculture | FAO | <http://www.fao.org/state-of-fisheries-aquaculture> |
| State of the World’s Forests | FAO | <https://www.unsystem.org/content/fao-state-worlds-forests-sofo> |
| State of the World’s Plants and Fungi | Royal Botanic Gardens Kew | <https://stateoftheworldsfungi.org/>  https://www.kew.org/science/state-of-the-worlds-plants-and-fungi |
| State of the World’s Sea Turtles Report | SWOT - State of the World’s Sea Turtles | <https://www.seaturtlestatus.org/swot-report> |
| State of the World’s Water | WaterAid | <https://washmatters.wateraid.org/publications/the-water-gap-state-of-the-worlds-water> |
| Status and Trends of Coral Reefs | Global Coral Reef Monitoring Network | <https://www.icriforum.org/document-types/icri-publications-reports-and-posters/> |
| TRAFFIC Briefing | TRAFFIC (IUCN & WWF) | <https://www.traffic.org/publications/> |
| Waterbird Population Estimates | Wetlands International | <http://wpe.wetlands.org/Imaps> |
| World Ocean Review | Maribus | <https://worldoceanreview.com/en/download/> |
| World Resources Report | WRI | <https://www.wri.org/our-work/project/world-resources-report/wrr> |
